# Compound jetting from bubble bursting at an air-oil-water interface

**DOI:** 10.1038/s41467-021-26382-w

**Published:** 2021-11-02

**Authors:** Bingqiang Ji, Zhengyu Yang, Jie Feng

**Affiliations:** 1 Department of Mechanical Science and Engineering, University of Illinois at Urbana-Champaign, Urbana, IL 61801 USA; 2 Materials Research Laboratory, University of Illinois at Urbana-Champaign, Urbana, IL 61801 USA

**Keywords:** Environmental impact, Rheology, Fluid dynamics

## Abstract

Bursting of bubbles at a liquid surface is ubiquitous in a wide range of physical, biological, and geological phenomena, as a key source of aerosol droplets for mass transport across the interface. However, how a structurally complex interface, widely present in nature, mediates the bursting process remains largely unknown. Here, we document the bubble-bursting jet dynamics at an oil-covered aqueous surface, which typifies the sea surface microlayer as well as an oil spill on the ocean. The jet tip radius and velocity are altered with even a thin oil layer, and oily aerosol droplets are produced. We provide evidence that the coupling of oil spreading and cavity collapse dynamics results in a multi-phase jet and the follow-up droplet size change. The oil spreading influences the effective viscous damping, and scaling laws are proposed to quantify the jetting dynamics. Our study not only advances the fundamental understanding of bubble bursting dynamics, but also may shed light on the airborne transmission of organic matters in nature related to aerosol production.

## Abstract (web summary)

How structurally complex interfaces mediate bubble bursting might significantly impact environmental and industrial processes. Here, authors investigate the bubble-bursting jets dynamics of oil-covered aqueous surface and show how these can also disperse insoluble organic contaminants.

## Introduction

Bubbles are widely observed at liquid surfaces, as they are easily formed by natural processes, such as wave breaking, raindrop impacts, and even changes of the solubility of air in water due to weather and seasons^1^. These surface bubbles eventually burst once the liquid film that forms the bubble cap drains to rupture, generating film droplets by cap disintegration, as well as jet droplets by fragmentation of the upwardly directed liquid jet formed at the end of cavity collapse^2^. These small droplets can remain suspended in the air, and therefore play a key role in mediating the mass transport across the interface in a wide range of physical, geological, and biological phenomena, including the flavor release from sparkling drinks^3^, sea spray aerosol generation for climate^4–8^, and even facilitation of vegetative reproduction over the ocean^9^.

Recently, bubbles have also been recognized as a major public health concern, regarding the aerosolization of contaminated water with harmful substances for airborne transmission of pollutants and infectious diseases^10,11^. In this context, the interplay between bubbles and a contaminated interface is a crucial factor for the aerosol droplet generation. In essence, rising bubbles scavenge surface-active organic material from the water column at their interfaces, and this process facilitates the formation of an organic-enriched air–liquid interface^12,13^. Examples include the sea-surface microlayer on the ocean, or the microorganism-enriched interface in the reaction tanks of municipal waste-water treatment plants^14,15^. Although some recent studies show that bubble bursting can disperse the surface organic layer as submicrometer droplets into water^16,17^ or eject organic matter into the atmosphere through film droplets^18,19^, the capability of such a chemically complex interface to manipulate the bubble physics, particularly the jetting, remains largely unknown. In particular, jet droplets play an important role in the mass flux of bubble bursting aerosols^4^. For instance, it has been found that jet droplets produce up to 43% of total submicrometer sea spray aerosol number concentrations^15^.

Here, we investigate the bubble bursting dynamics at an oil-covered aqueous surface by high-speed imaging, to characterize the previously unknown interplay between bubbles and a structurally complex interface with an insoluble viscous oil layer. We show that a compound multiphase jet is formed, resulting from the spreading of the oil layer on the air-water surface of the bubble cavity in a complete-wetting state. We found that even highly viscous oil can be ejected into the atmosphere with the compound jetting, where the oil layer viscosity is far beyond the maximum viscous limit for a bubble bursting jet to produce droplets in a single liquid. By modeling the extra viscous dissipation regarding the oil film spreading along the cavity surface, we propose scaling laws that quantify the contribution of the oil viscosity and oil layer thickness to the change in the radius and velocity of the jet tip. Our results provide potential guidance for the prediction of organic-enriched aerosol produced by bubble bursting at a structurally complex interface.

## Results

### High-speed imaging of bubble bursting

**Fig. 1:**
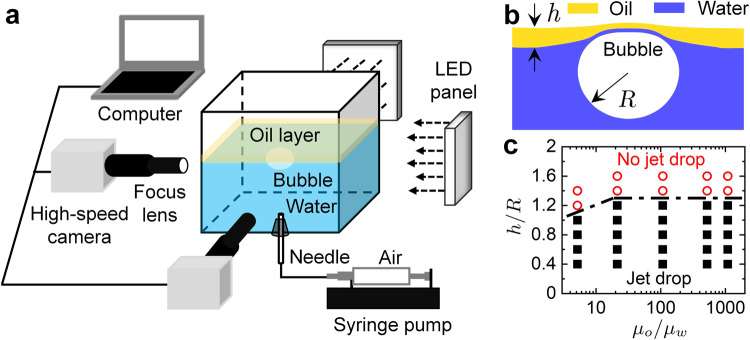
Experimental setup, sketch of a still bubble at an oil-covered aqueous surface and regime map of bubble bursting jets. **a** Schematic of the experimental apparatus. The air bubble with an equivalent radius of *R* = 1.67 mm is generated by a needle at the bottom of the container with a syringe pump, and it rises to the water surface covered by an oil layer with a thickness *h*. The bubble rests at the oil-covered aqueous surface, and then bursts, which is recorded by two computer-controlled synchronized high-speed cameras with LED illumination. **b** Close-up sketch of an air bubble with radius *R* resting at an aqueous surface covered by an oil layer with a thickness *h*, which typifies an organic-enriched, structurally complex interface. **c** Regime map of bubble bursting jets at an oil-covered aqueous surface regarding the oil viscosity *μ*_o_ and the oil layer thickness *h*, showing the boundary between no jet droplet and jet droplet of the bubble bursting. Jet droplets only occur when the oil layer thickness is smaller than a critical thickness *h*_c_, where the oil and water layers rupture simultaneously and oily jet droplets are ejected into air. When *h* > *h*_c_, the water film ruptures first and then the whole bubble enters the oil layer, and the subsequent bubble bursting jet does not eject any oily aerosol droplets (Supplementary Fig. 1 and Supplementary Movies 1–3).

The bubble bursting dynamics at an oil-covered aqueous surface is observed by two synchronized high-speed cameras from side views above and below the liquid surface (Fig. 1a). No surface-active material is added in any liquid. The gas bubble approaches the oil-covered aqueous surface after being released. Then the bubble rests at the air–oil–water interface with a cap consisting of water and oil films after its initial oscillation has died down (Fig. 1b), and finally bursts. The bubble shape at rest is almost unchanged in the pure water and different oily cases. We fixed the equivalent radius of the bubble as *R* = 1.67 mm and used 5–1000 cSt silicone oils in our experiments (Table 1). When the oil layer thickness *h* is larger than a critical thickness *h*_c_ (≈1.2*R*), the water film on the bubble cap ruptures first, and subsequently the whole bubble enters the oil layer. In such a regime, no jet droplets are emitted by bubble bursting at the viscous oil layer in the experiments (Supplementary Fig. 1 and Supplementary Movies 1–3). While for *h* ≤ *h*_c_, the oil and water films on the bubble cap rupture simultaneously, and the subsequent cavity collapse results in a compound multiphase jet that ejected droplets. Therefore, all experiments were performed with *h* ≤ *h*_c_ (Fig. 1c).

**Table 1:** Physical properties of the liquids used in the experiments.

Liquids	*ρ* (kg/m^3^)	*μ* (mPa ⋅ s)	*γ*_wa_ (mN/m)	*γ*_oa_ (mN/m)	*γ*_ow_ (mN/m)
DI water	998	0.89	71.6 ± 1.0	N/A	N/A
5 cSt silicone oil	913	4.6	N/A	18.7 ± 0.3	38.1 ± 0.4
20 cSt silicone oil	950	19	N/A	19.4 ± 0.7	40.9 ± 0.5
100 cSt silicone oil	960	96	N/A	20.1 ± 0.2	43.7 ± 0.4
500 cSt silicone oil	970	465	N/A	19.9 ± 0.1	38.7 ± 0.2
1000 cSt silicone oil	970	970	N/A	21.7 ± 0.2	40.8 ± 1.0

*ρ* density, *μ* dynamic viscosity, *γ*_wa_ surface tension of water, *γ*_oa_ surface tension of oil, *γ*_ow_ oil–water interfacial tension.

### Oily jetting

**Fig. 2:**
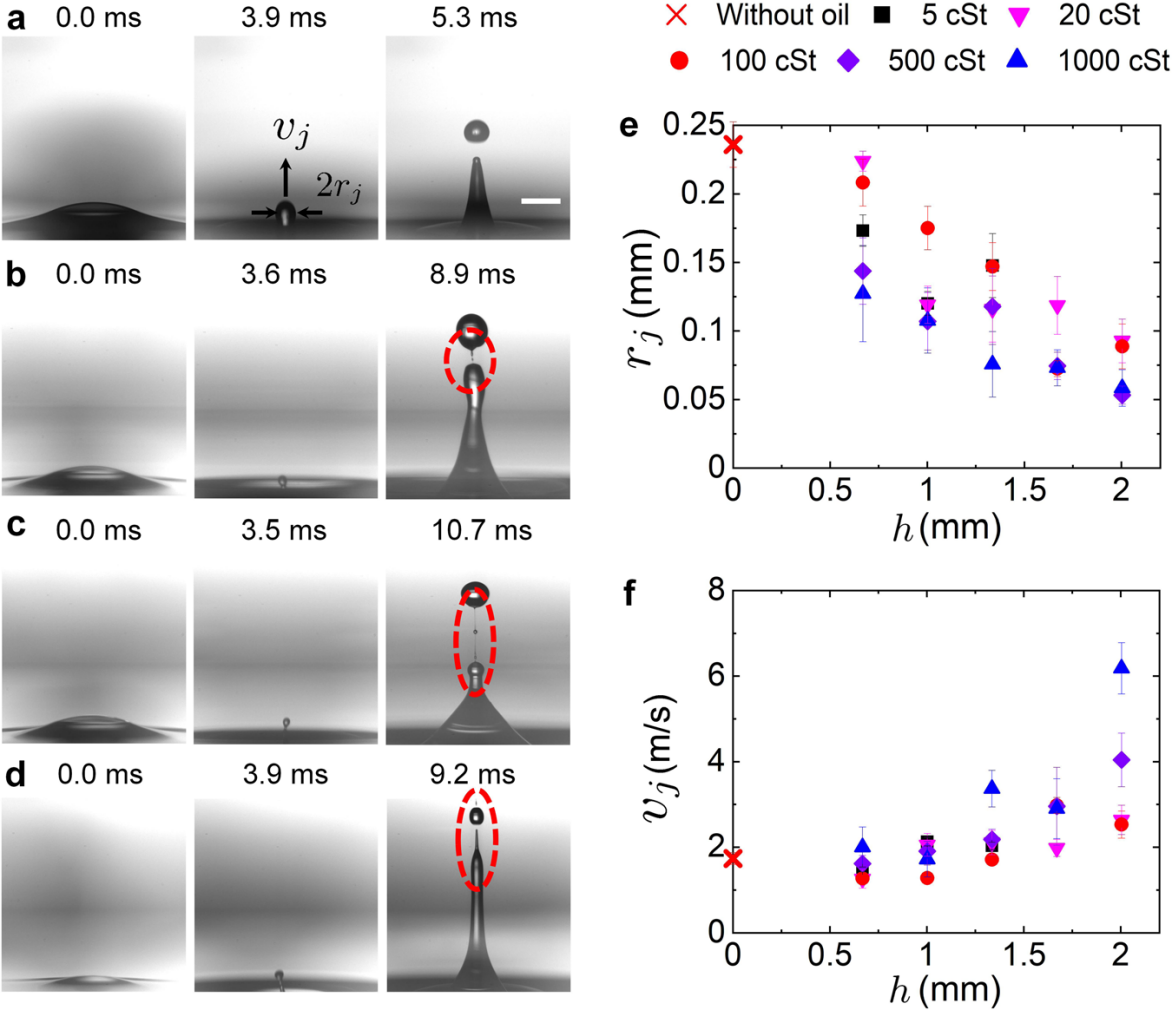
Snapshots of bubble bursting jetting and the measured jet tip radius and velocity. Side-view snapshots of jet formation during bubble bursting at the aqueous surface (**a**) without an oil layer, and covered by a layer of (**b**) 5 cSt silicone oil, *h*/*R* = 0.6, (**c**) 500 cSt silicone oil, *h*/*R* = 0.6, and (**d**) 500 cSt silicone oil, *h*/*R* = 1.2. *R* = 1.67 mm in the experiments. Scale bar = 1 mm. *t* = 0 represents the beginning of bubble bursting when a hole nucleates in the bubble cap. An oily jet is generated (column 2 of **a**–**d**) following the cavity collapse, which becomes smaller but faster with the increasing viscosity and thickness of the oil layer. A thin oily thread is observed in all oily cases (highlighted in column 3 of **b**–**d**). Dependencies of (**e**) the jet tip radius *r*_j_ and (**f**) jet tip velocity *v*_j_ on the oil layer thickness *h* for different oil viscosities. *r*_j_ and *v*_j_ are measured when the jet tip crosses the undisturbed air–oil interface. Error bars are calculated as the standard deviations of data of at least 10 runs.

In our experimental system, after the simultaneous rupture of the oil and water films on the bubble cap, the cavity collapses, with capillary waves propagating downwards and finally focusing at the cavity nadir. Then the curvature of the interface at the bubble nadir reverses, generating an upward Worthington jet^20^ that finally breaks up due to Rayleigh-Plateau instability and disperses myriads of droplets, known as jet droplets, into air. Surprisingly, unlike the bubble bursting jet from a clean water surface^3,21,22^, a long and thin liquid thread appears during the jet pinch-off from an oil-covered aqueous surface (Fig. 2b–d). Such a feature is a typical characteristic of highly viscous fluid pinch-off^23,24^, which indicates the formation of an oily jet. In addition, we observed dumbbell-shaped jet droplets produced by such a compound jet (Supplementary Fig. 2 and Supplementary Movie 4), which is another distinct feature compared with bubble bursting at a clean water surface. These droplets remain uncoalesced until they fall onto the oil layer with a much longer life time compared with pure water droplets. We believe the oil coating delays the droplet coalescence, as a further evidence for the compound droplets from the oily jetting. In addition, by dispersing tracer microparticles into the oil layer and imaging the particles from the dried top jet droplet collected on a glass slide, we directly evidenced the presence of oil in the jet droplets (Supplementary Fig. 3), and the top jet droplet could contain an oil volume fraction of up to 83% (see SI for the calculation).

It has been well established that the jet formation after the cavity collapse controls the follow-up droplet generation, including the jet droplet size and velocity, as shown in prior work for bubble bursting at a single liquid surface^25,26^. In particular, the jet tip radius and velocity show similar trends as the top jet droplet radius and velocity^26^. Therefore, we mainly focus on characterizing the jet dynamics in the current work, as a first step to inform the interplay between bubbles and a compound interface. The radius, *r*_j_, and velocity, *v*_j_, of the jet tip are measured when the tip crosses the horizontal level of undisturbed air–oil interface (Fig. 2a). Remarkably, we observe that the oily jet is thinner and travels faster, i.e., *r*_j_ decreases and *v*_j_ increases with increasing oil viscosity (*μ*_o_) and oil layer thickness (*h*) (Fig. 2e, f), and eventually smaller oily jet droplets are ejected with a faster speed compared with the case of an air-water surface (Fig. 2a–d). The radius and velocity of the top jet droplet at the moment of detachment are also measured, as shown in Supplementary Fig. 4. Meanwhile, both the jetting height and jet droplet number increase with *μ*_o_ and *h* (Supplementary Movie 5).

Previous studies show that the jet dynamics of bubble bursting at a clean liquid surface is mainly controlled by two dimensionless numbers, e.g., the Bond number Bo = *ρgR*^2^/*γ* and Ohnesorge number

1 $${{{{{\mathrm{Oh}}}}}}=\frac{\mu }{\sqrt{\rho \gamma R}},$$

where *ρ*, *μ*, *γ* indicate liquid density, dynamic viscosity, and interfacial tension, respectively, and *g* is the gravitational acceleration^21,25,27^. Under conditions in which Bo (effect of gravity relative to surface tension) exceeds 3, no jet droplets are produced because of the influence of gravity on the cavity shape. Similarly, when Oh (effect of viscosity to inertial and surface tension) exceeds 0.03–0.04, jet droplets are not produced since viscous stresses suppress jetting by damping out the inertial capillary waves driving the motion^28,29^.

Using the silicone oil properties in our current experiments (Table 1), Bo = 1.2−1.4 and Oh = 0.03−5, no jet droplet is expected by bubble bursting at the surface of a bulk silicone oil^28^. However, the ejection of the oily jet droplet is observed in our experiment, even if the oil layer viscosity is high. Therefore, for a compound interface with a viscous oil layer on top of the water surface, bubble bursting jet can actually eject droplets containing the very viscous oil component as shown by our study. This feature is not predicted by the maximum viscous limit, considering Oh, for jet droplets to form at a clean bulk liquid surface in prior work. This indicates that for an organic-enriched interface, such as the sea-surface microlayer, the highly viscous surface organic matter may be ejected into the atmosphere through the break-up of a bubble bursting jet, raising the question of how the oils at the aqueous surface transport to the jet and jet droplets produced by a clean gas bubble.

### Oil spreading on cavity surface

**Fig. 3:**
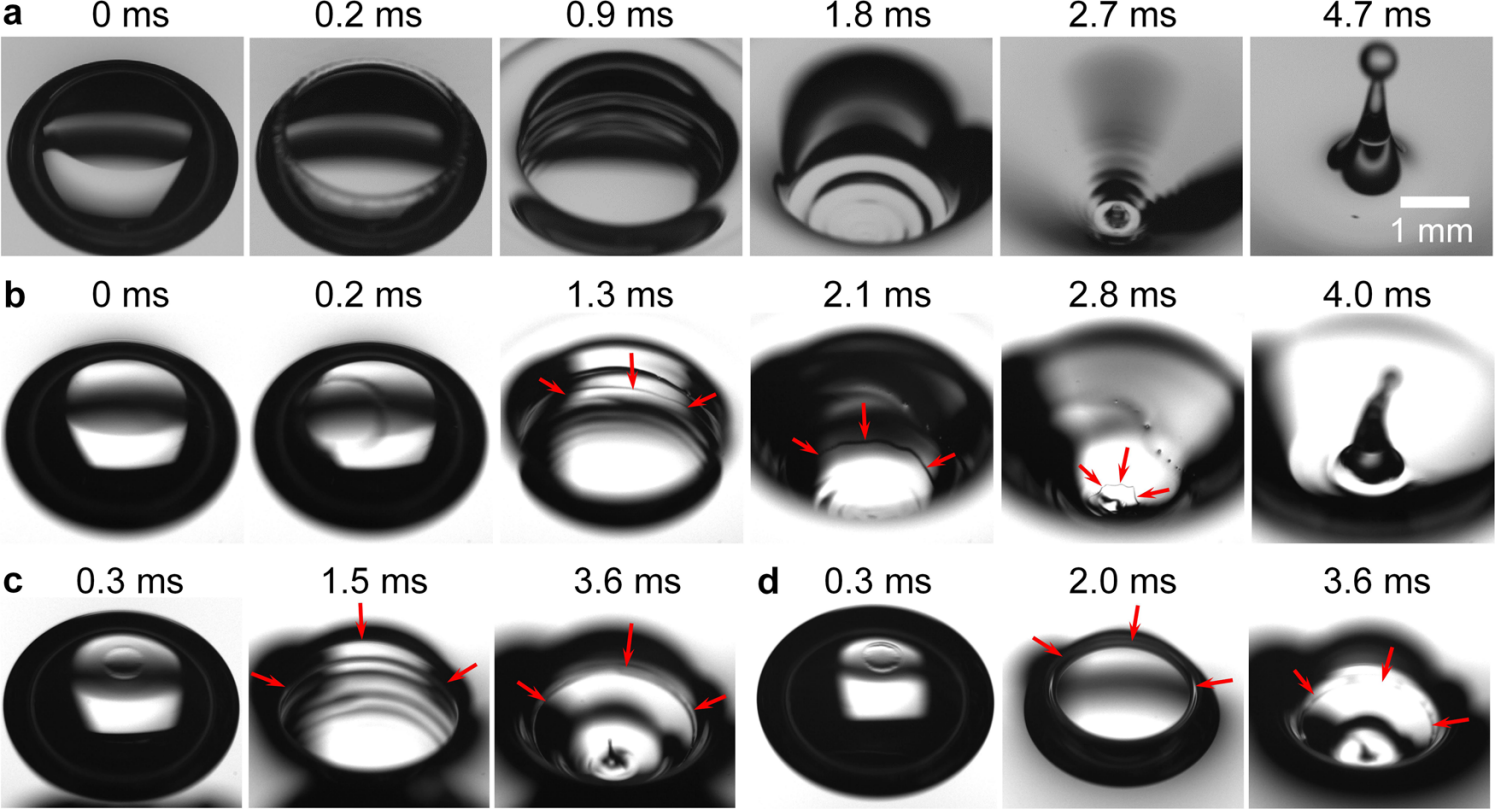
Top-view snapshots of oil spreading on the cavity surface during bubble bursting at the aqueous surface. The water surfaces shown here are (**a**) clean, and covered by a layer of (**b**) 100 cSt silicone oil, *h*/*R* = 0.8, (**c**) 1000 cSt silicone oil, *h*/*R* = 0.8, and (**d**) 1000 cSt silicone oil, *h*/*R* = 1.2. Scale bar = 1 mm. Comparing with the pure water case (**a**), a macroscopic oil edge (marked by red arrows) along the cavity after the cap rupture at an oil-covered aqueous surface is clearly observed, which is generated by oil spreading in the complete-wetting state (**b**–**d**). The macroscopic oil edges can be distinguished since they typically show asymmetric and irregular morphology, while the capillary waves from cavity collapse show axisymmetric and smooth patterns. Additional evidence is shown in Supplementary Fig. 5 with tracer particles to assist visualization of the macroscopic oil edges.

Here we show that the formation of oily jets results from the spreading of oils along the surface of the collapsing cavity. For the bubble dynamics at an air–oil–water interface, it is important to consider physicochemical interaction between oil and water molecules, as highlighted by our previous work about nanoemulsions produced by bubble bursting^16,30^. The bubble cavity surface below the oil–water interface before bubble bursting is initially oil-free as shown in Fig. 1b. Given the positive spreading coefficient Δ*γ* = *γ*_wa_ − *γ*_oa_ − *γ*_ow_ > 0 (Table 1) in the complete-wetting state between silicone oil and water, the oil spontaneously spreads along the cavity surface towards the bubble nadir once the cap ruptures. In addition, it has been shown by previous studies^31,32^ that the spreading of a silicone oil droplet on an aqueous surface exhibits two distinct spreading fronts with non-uniform thicknesses, i.e., a faster microscopic front connecting to a slower macroscopic bulk edge. From the high-speed imaging as shown in Fig. 3 and Supplementary Fig. 5, a macroscopic oil edge propagating along the cavity surface is observed. In addition, the propagation of the macroscopic spreading edge is slower with increasing oil viscosity (Fig. 3b, c) but basically unaffected by the oil layer thickness (Fig. 3c, d). For instance, the macroscopic spreading edge of 100 cSt oil arrives at the cavity nadir at the onset of jet formation (*t* = 2.8 ms in Fig. 3b), and that of 1000 cSt oil is still far away from the cavity nadir (*t* = 3.6 ms in Fig. 3c, d) when the nadir curvature reverses.

While we cannot directly visualize the microscopic front ahead of the macroscopic oil edge since the adjacent oil-film thickness is at the order of *O*(1 nm)^33^, the surface-tension-driven spreading speed of the microscopic oil front can be estimated by balancing the net driving force and the viscous force that developed at the boundary layer of the supporting fluid. Then the time for the microscopic oil front reaching the bubble cavity nadir can be estimated as^31,32,34^

2 $${t}_{{{{{{\mathrm{s}}}}}}} \sim {\left(\frac{{\mu }_{{{{{{\mathrm{w}}}}}}}{\rho }_{{{{{{\mathrm{w}}}}}}}{R}^{4}}{{{\Delta }}{\gamma }^{2}}\right)}^{1/3},$$

while the time of cavity collapse can be written as the capillary time^25,35^ calculated based on the effective surface tension of the air–oil–water interface *γ*_e_ = *γ*_oa_ + *γ*_ow_^36^,

3 $${t}_{{{{{{\mathrm{c}}}}}}} \sim {\left(\frac{{\rho }_{{{{{{\mathrm{w}}}}}}}{R}^{3}}{{\gamma }_{{{{{{\mathrm{e}}}}}}}}\right)}^{1/2}.$$

In our experiments, we find *t*_s_ ≈ *t*_c_ ≈ *O*(1 ms). The comparable time scales indicate that the microscopic oil front is able to reach the base of the cavity and the compound jet can form, confirming our observations. In addition, we find that a more viscous substrate liquid than water weakened the effect of the oil layer on the jet dynamics (Supplementary Fig. 6), presumably due to the slower spreading of the oil film (larger *t*_s_) on the cavity surface, showing the important role of the oil spreading dynamics.

### Cavity collapse and capillary wave propagation

**Fig. 4:**
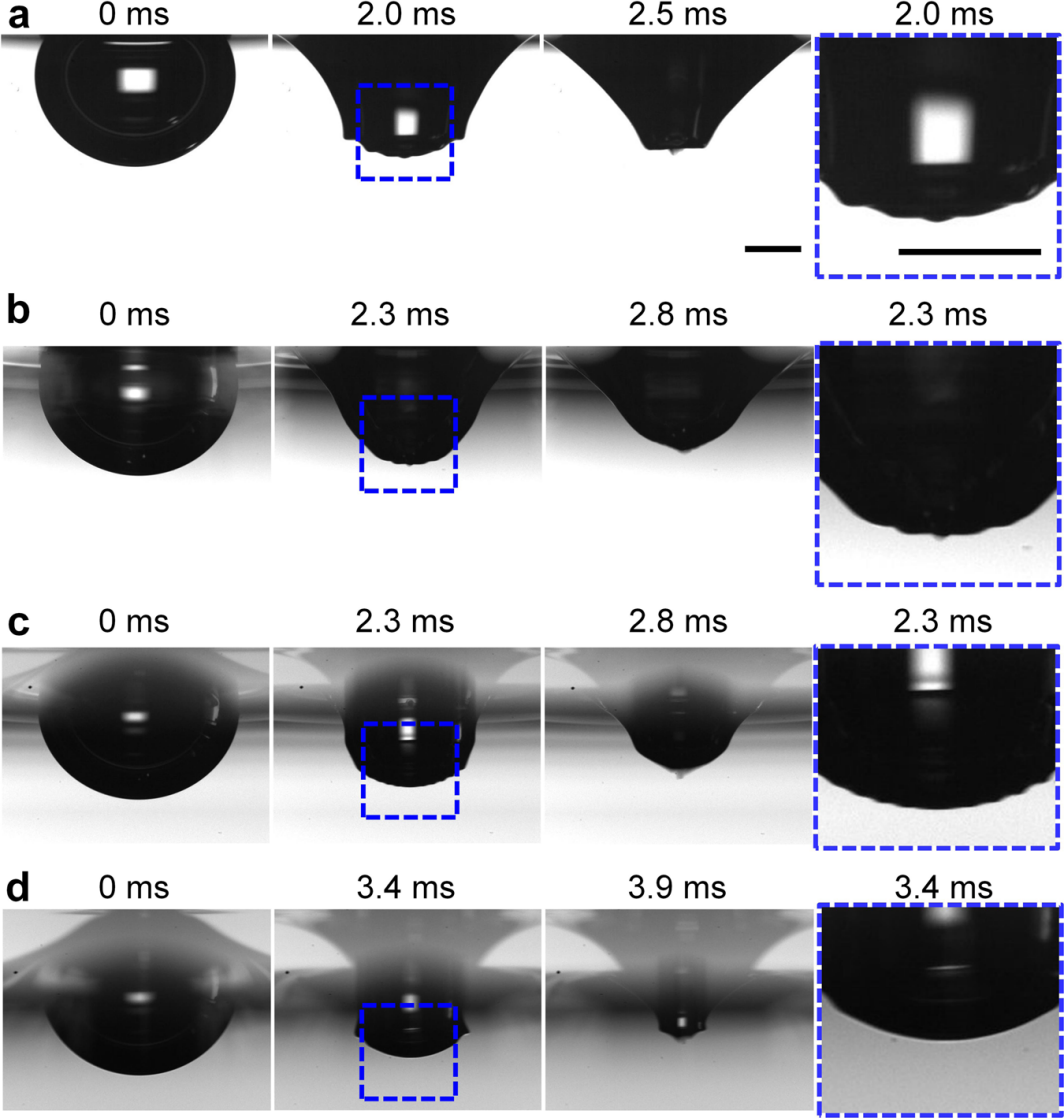
Side-view snapshots of cavity collapse during bubble bursting. The water surfaces shown here are (**a**) clean, or covered by a layer of (**b**) 100 cSt silicone oil, *h*/*R* = 0.8, (**c**) 1000 cSt silicone oil, *h*/*R* = 0.8, and (**d**) 1000 cSt silicone oil, *h*/*R* = 1.2. The equivalent bubble radius *R* = 1.67 mm in the experiments. Scale bars represent 1 mm. The capillary waves during cavity collapse are progressively damped in the oily cases compared with those in the pure water case (column 4 as the zoom-in images of the blue square zones in column 2), which results in a sharper tip at the cavity nadir in the oily cases (column 3), indicating the additional damping effect from the oil layer.

We further investigate how the oil spreading at the cavity surface modifies the jet dynamics. It has been stated that the capillary wave propagation during cavity collapse plays an important role in the jet dynamics produced by bubble bursting. During cavity collapse, the rim retraction after the bubble cap rupture excites a series of capillary waves propagating along the cavity surface (Fig. 4). The capillary waves with relatively smaller wavelengths travel faster and are more rapidly attenuated by viscosity^25,27^. The dominant capillary wave selected by viscous attenuation, i.e., the capillary wave with the shortest wavelength not attenuated by viscosity during cavity collapse, can successfully propagate to the bubble nadir and makes the cavity collapse to a truncated cone, which triggers the ejection of a jet^25^.

**Fig. 5:**
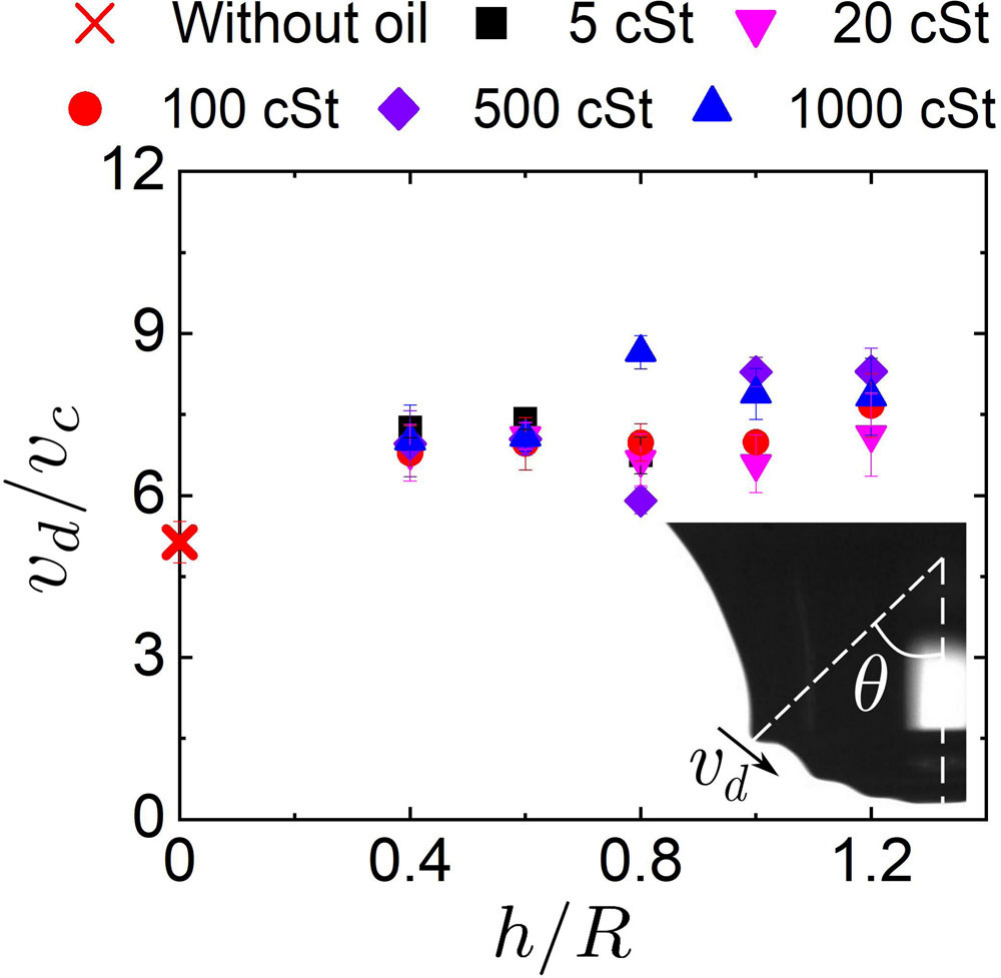
Capillary wave velocity. Dependence of dimensionless velocity *v*_d_/*v*_c_ of the selected dominant capillary wave on the dimensionless oil layer thickness *h*/*R* during bubble bursting at the oil-covered aqueous surface, where ${v}_{{{{{{\mathrm{c}}}}}}}={\left(\frac{{\gamma }_{{{{{{\mathrm{e}}}}}}}}{{\rho }_{{{{{{\mathrm{w}}}}}}}R}\right)}^{1/2}$ is the inertial capillary velocity. Inset shows that *v*_d_ is measured when the selected dominant wave goes through the position with an angle of *θ* = *π*/4 corresponding to the vertical cavity axis. Error bars are calculated as the standard deviations of data of at least 10 runs.

Considering the essential role of capillary wave dynamics, we measured the selected dominant wave speed, *v*_d_, as the propagation speed when the last wave crest crosses the position with an angle of *π*/4 corresponding to the vertical cavity axis following the previous study^25^. We found that *v*_d_/*v*_c_ = 7.2 ± 1.3 which is almost unaffected by the oil layer (Fig. 5), with the inertial capillary velocity *v*_c_ calculated based on *γ*_e_ as

4 $${v}_{{{{{{\mathrm{c}}}}}}}={\left(\frac{{\gamma }_{{{{{{\mathrm{e}}}}}}}}{{\rho }_{{{{{{\mathrm{w}}}}}}}R}\right)}^{1/2}.$$

This velocity ratio is consistent with that in previous numerical study on bubble bursting jetting at a clean liquid surface as *v*_d_/*v*_c_ ≈ 5, which is independent of Oh^37^. The wavelength of the selected dominant wave, *λ*_d_, which was suggested to be estimated as the minimum radii of curvature of the capillary waves, was found to increase with Oh due to the larger viscous attenuation^25,37^. An increasing *λ*_d_ can shorten the lower base of the truncated cone-shaped cavity, and hence decreases the jet radius and increases the jet velocity when Oh < 0.03 for bubble bursting at a clean liquid surface^25,26,37^. Though we could not precisely measure the radii of curvature of the capillary waves due to the spatial resolution limit of the experimental images, a smaller radius at the lower base of the truncated cone-shaped cavity is observed at the moment of jet formation when increasing the oil viscosity and layer thickness (column 3 in Fig. 4), suggesting a larger *λ*_d_. Meanwhile, we found that while the bubble cavity shapes remain similar, the amplitudes of the capillary waves are noticeably damped by the presence of oil (Fig. 4), and the damping effect increases with *μ*_o_ and *h*. With increasing oil viscosity and layer thickness, more capillary waves with small wavelengths are completely damped (columns 2, 4 in Fig. 4) and the cavity profile becomes smoother. The similar trend has also been reported by previous experimental studies, which argued that with increasing Oh, the capillary waves are progressively damped, resulting in a smaller and faster jet^3,21,22^.

From the above observations on the cavity profile as well as the capillary wave propagation, the viscous damping of the capillary waves as well as the wavelength of the selected dominant wave is increased with the oil viscosity and layer thickness. Combining our argument on oil spreading at the cavity surface, we expect that the viscous attenuation and selection of the capillary waves are modified by the presence of a more viscous oil film on the bubble cavity surface, and the viscous damping rate increases with oil viscosity and layer thickness. Next, we will explore how to connect such damping on the jet ejection with the oil spreading.

### Viscous damping with oil spreading

While Bo compares the gravity and capillary effects, Oh compares the viscous damping rate of the cavity collapse, ${t}_{{{{{{\mathrm{d}}}}}}}^{-1}$, and the inverse of the time of cavity collapse, ${t}_{{{{{{\mathrm{c}}}}}}}^{-1}$^25,35^. Our experimental observation shows that the capillary waves are progressively damped with the increase of the oil viscosity and layer thickness, indicating a higher viscous damping rate of cavity collapse. Here we derive the viscous damping rate with an oil-covered interface combining the oil spreading dynamics.

**Fig. 6:**
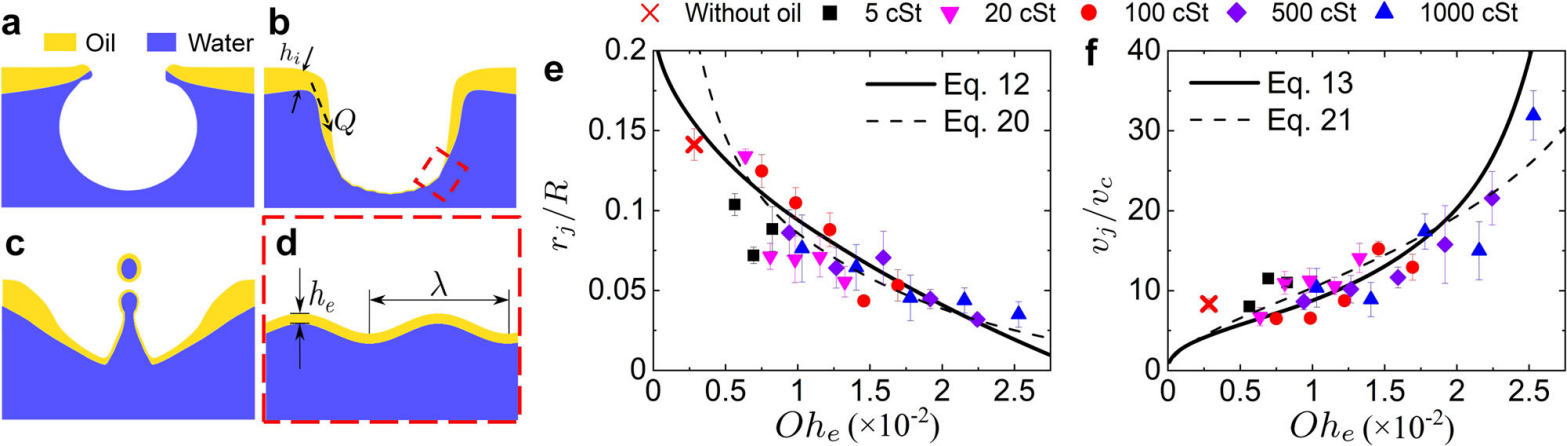
Schematics of bubble bursting at an air–oil–water interface and scaling laws of jet tip radius and velocity. **a** Cap rupture, **b** cavity collapse, and **c** jet formation of bubble bursting at an oil-covered aqueous surface. The oil spreading along the cavity surface has a flow rate of *Q* from the oil layer around the cavity with an initial thickness of *h*_i_. **d** Zoom-in view of the red square zone in (**b**), showing a capillary wave of wavelength *λ* at a water surface covered with an oil film of thickness *h*_e_. Dimensionless (**e**) radius *r*_j_/*R* and (**f**) velocity *v*_j_/*v*_c_ of the jet tip as a function of the revised Ohnesorge number Oh_e_. In (**e**) and (**f**), the solid lines represent the scaling laws of Eqs. (12) and (13), and the dashed lines represent the scaling laws of Eqs. (20) and (21), respectively. Error bars are calculated as the standard deviations of data of at least 10 runs.

The thin oil film spreading along the cavity surface can be considered as a viscous surface film with two-dimensional shear viscosity *μ*_o_*h*_e_ and dilatational viscosity 3*μ*_o_*h*_e_^38^, where *h*_e_ is the film thickness (see Fig. 6d and ‘Methods’ for the detailed derivation). Thus, for such a compound interface, we obtain a leading order approximation for the total dissipation rate of a capillary wave with a wavelength *λ* and wave number *k* ~ *λ*^−1^ as

5 $$\langle D\rangle =\frac{4({\mu }_{{{{{{\mathrm{w}}}}}}}+{\mu }_{{{{{{\mathrm{o}}}}}}}k{h}_{{{{{{\mathrm{e}}}}}}}){k}^{2}}{{\rho }_{{{{{{\mathrm{w}}}}}}}}{E}_{0}.$$

Since the amplitude of capillary waves, *α* decreases exponentially in time by ${{{{{{{{\rm{e}}}}}}}}}^{-t/{t}_{{{{{{\mathrm{d}}}}}}\lambda }}$^39^, while ${E}_{0}\propto {\alpha }^{2}\propto {{{{{{{{\rm{e}}}}}}}}}^{-2t/{t}_{{{{{{\mathrm{d}}}}}}\lambda }}$ with ${t}_{{{{{{\mathrm{d}}}}}}\lambda }^{-1}$ as the effective damping rate of the capillary wave with a wavelength *λ*, we consider

6 $$\frac{{{{{{{{\rm{d}}}}}}}}{E}_{0}}{{{{{{{{\rm{d}}}}}}}}t}=-\langle D\rangle .$$

Therefore, we obtain

7 $${t}_{{{{{{\mathrm{d}}}}}}\lambda }^{-1}=2\frac{{\mu }_{{{{{{\mathrm{w}}}}}}}+{\mu }_{{{{{{\mathrm{o}}}}}}}k{h}_{{{{{{\mathrm{e}}}}}}}}{{\rho }_{{{{{{\mathrm{w}}}}}}}}{k}^{2}.$$

We note that Eq. (7) also agrees with the expression for the capillary wave damping rate due to a viscous surface fluid layer by Jenkins and Jacobs^36^. Though there is a series of capillary waves with different *λ* propagating along the cavity surface, the only characteristic length of the initial condition of the system, *R*, is adopted in ${t}_{{{{{{\mathrm{d}}}}}}}^{-1}$ which characterizes the viscous damping of the cavity collapse^25,35,40^. Following this argument, by using *k* ~ *R*^−1^ in our experiment, we further obtain

8 $${t}_{{{{{{\mathrm{d}}}}}}}^{-1} \sim \frac{{\mu }_{{{{{{\mathrm{w}}}}}}}+\frac{{h}_{{{{{{\mathrm{e}}}}}}}}{R}{\mu }_{{{{{{\mathrm{o}}}}}}}}{{\rho }_{{{{{{\mathrm{w}}}}}}}{R}^{2}}.$$

We observe that the oil film spreading along the cavity surface has a non-uniform thickness profile (Fig. 3b–d). For the sake of simplicity to reveal the driving physical mechanism, we approximate the average film thickness *h*_e_ with respect to the effective damping rate, which is assumed to be mainly contributed by the spreading of the macroscopic oil film considering the much smaller thickness of the precursor film in comparison. For an oil layer surrounding the bubble cavity, we consider the layer as an infinite reservoir localized at the cavity edge, which provides a flow rate *Q* to form the oil film on the bubble cavity. *Q* is estimated as $Q\, \sim \,2\pi R{h}_{{{{{{\mathrm{i}}}}}}}\bar{v}$, where *h*_i_ is the initial oil layer thickness at the boundary of the bubble cavity before spreading (Fig. 6b), and $\bar{v}$ is the average inward spreading velocity of the macroscopic oil edge. Similar to the scenario for inward spreading of a healing capillary film^41^, the time evolution of *h*_i_ scales with the initial oil layer thickness *h*, while $\bar{v}$ is estimated by *γ*_e_/*μ*_o_^42,43^. Therefore, *Q* ~ 2*πRhγ*_e_/*μ*_o_, and the average oil thickness *h*_e_ ~ *Qt*_c_/*πR*^2^, where *t*_c_ is the time of cavity collapse as the capillary time (Eq. (3)). Now by scaling analysis, we obtain

9 $${h}_{{{{{{\mathrm{e}}}}}}}\, \sim \,{{{{{\mathrm{O}}}}}}{{{{{{\mathrm{h}}}}}}}_{{{{{{\mathrm{o}}}}}}}^{-1}h,$$

where ${{{{{\mathrm{O}}}}}}{{{{{{\mathrm{h}}}}}}}_{{{{{{\mathrm{o}}}}}}}={\mu }_{{{{{{\mathrm{o}}}}}}}/\sqrt{{\rho }_{{{{{{\mathrm{o}}}}}}}{\gamma }_{{{{{{\mathrm{e}}}}}}}R}$, showing that the oil film on the bubble cavity formed by inward spreading can be related to the initial oil layer thickness *h* by Oh_o_. Given the simplified configuration (Fig. 6b, d) in this study, we revise Eq. (9) as ${h}_{{{{{{\mathrm{e}}}}}}}\, \sim \,a{{{{{\mathrm{O}}}}}}{{{{{{\mathrm{h}}}}}}}_{{{{{{\mathrm{o}}}}}}}^{b}h$, with *a* and *b* as the numerical factors obtained from experiments. Thus, the effective viscous damping rate of the cavity collapse is written as

10 $${t}_{{{{{{\mathrm{d}}}}}}}^{-1} \sim \frac{{\mu }_{{{{{{\mathrm{w}}}}}}}+a{{{{{\mathrm{O}}}}}}{{{{{{\mathrm{h}}}}}}}_{{{{{{\mathrm{o}}}}}}}^{b}\frac{h}{R}{\mu }_{{{{{{\mathrm{o}}}}}}}}{{\rho }_{{{{{{\mathrm{w}}}}}}}{R}^{2}}.$$

### Scaling laws for the oily jetting

By modeling the dynamics of both bubble cavity collapse and oil spreading, we now propose scaling laws to predict *r*_j_ and *v*_j_ for the compound jetting. We note that Bo ≈ 0.4 was kept the same throughout all the experiments (Table 1). Meanwhile, the Oh can be revised adopting the effective viscous damping rate of the cavity collapse ${t}_{{{{{{\mathrm{d}}}}}}}^{-1}$ (Eq. (10)) for our experiments, yielding a revised Ohnesorge number as

11 $${{{{{\mathrm{O}}}}}}{{{{{{\mathrm{h}}}}}}}_{{{{{{\mathrm{e}}}}}}}=\frac{{t}_{{{{{{\mathrm{d}}}}}}}^{-1}}{{t}_{{{{{{\mathrm{c}}}}}}}^{-1}}=\frac{{\mu }_{{{{{{\mathrm{w}}}}}}}+a{{{{{\mathrm{O}}}}}}{{{{{{\mathrm{h}}}}}}}_{{{{{{\mathrm{o}}}}}}}^{b}\frac{h}{R}{\mu }_{{{{{{\mathrm{o}}}}}}}}{{({\rho }_{{{{{{\mathrm{w}}}}}}}{\gamma }_{{{{{{\mathrm{e}}}}}}}R)}^{1/2}}.$$

For bubble bursting at a clean liquid surface, *r*_j_ decreases and *v*_j_ increases monotonically with Oh when Oh < Oh_c_^22,25,40^, where Oh_c_ ≈ 0.0305^37^. Our experimental results show the same trends which means Oh_e_ < Oh_c_ (Fig. 2e, f). By balancing the viscous damping rate of the capillary wave ${t}_{{{{{{\mathrm{d}}}}}}\lambda }^{-1}$ calculated using Eq. (7) with ${t}_{{{{{{\mathrm{c}}}}}}}^{-1}$, the wavelength of the selected dominant wave can be obtained as ${\lambda }_{{{{{{\mathrm{d}}}}}}}/R\propto {{{{{\mathrm{O}}}}}}{{{{{{\mathrm{h}}}}}}}_{{{{{{\mathrm{e}}}}}}}^{1/2}$, similar to the argument for bubble bursting at a clean liquid surface with Oh < Oh_c_^25^. It was suggested that *λ*_d_ sets the radius at the base of the truncated cone-shaped cavity at the onset of jet formation, which further determines the radius of the jet tip, yielding ${r}_{{{{{{\mathrm{j}}}}}}}/R={c}_{r}(1-{({{{{{\mathrm{Oh}}}}}}/{{{{{\mathrm{O}}}}}}{{{{{{\mathrm{h}}}}}}}_{{{{{{\mathrm{c}}}}}}})}^{1/2})$ with Oh < Oh_c_^37^. Therefore, with the revised Ohnesorge number Oh_e_, we propose that

12 $$\frac{{r}_{{{{{{\mathrm{j}}}}}}}}{R}={c}_{r}\left(1-{\left(\frac{{{{{{\mathrm{O}}}}}}{{{{{{\mathrm{h}}}}}}}_{{{{{{\mathrm{e}}}}}}}}{{{{{{\mathrm{O}}}}}}{{{{{{\mathrm{h}}}}}}}_{{{{{{\mathrm{c}}}}}}}}\right)}^{1/2}\right),\,{{\mbox{for}}}\,{{{{{\mathrm{O}}}}}}{{{{{{\mathrm{h}}}}}}}_{{{{{{\mathrm{e}}}}}}} \, < \, {{{{{\mathrm{O}}}}}}{{{{{{\mathrm{h}}}}}}}_{{{{{{\mathrm{c}}}}}}}{{\mbox{}}},$$

where *c*_*r*_ = 0.2215 as predicted by the prior work^37^. For bubble bursting at a clean liquid surface, prior work also argued that *v*_j_ can be calculated as the flow generated by a continuous line of sinks extending along the axis of symmetry a distance proportional to *λ*_d_^37^. Based on this argument, we further propose that

13 $$\frac{{v}_{{{{{{\mathrm{j}}}}}}}}{{v}_{{{{{{\mathrm{c}}}}}}}}=\frac{{c}_{v}}{{(-{{\mbox{ln}}}({r}_{{{{{{\mathrm{j}}}}}}}/R))}^{1/2}}\left(\frac{1}{{r}_{{{{{{\mathrm{j}}}}}}}/R}-\frac{1}{{r}_{{{{{{\mathrm{j}}}}}}}/R+4.67{{{{{\mathrm{O}}}}}}{{{{{{\mathrm{h}}}}}}}_{{{{{{\mathrm{e}}}}}}}^{1/2}}\right),{{\mbox{for}}}\,{{{{{\mathrm{O}}}}}}{{{{{{\mathrm{h}}}}}}}_{{{{{{\mathrm{e}}}}}}} \, < \, {{{{{\mathrm{O}}}}}}{{{{{{\mathrm{h}}}}}}}_{{{{{{\mathrm{c}}}}}}}{{\mbox{}}},$$

where *r*_j_/*R* is calculated by Eq. (12), and *c*_*v*_ is found to be ≈1.5. The smaller *c*_*v*_ compared to the literature value of 4.2^37^ may be attributed to the deceleration of the jet tip when it rises from the bubble nadir to the surface^37^ and the effect of relatively large Bo^40^ in the current experiments.

For a wide range of experimental conditions, including viscosity of 5–1000 cSt and layer thickness of 0.67–2 mm, we find that Eqs. (12) and (13) agree well with our experimental results as shown in Fig. 6e, f. Here, Oh_e_ takes into account the additional viscous dissipation by the oil layer film on the cavity surface formed by spontaneous spreading, related to the oil viscosity and initial layer thickness. We note that *a* = 0.015 and *b* = −0.8 in Eq. (11) obtained by experimental fitting, indicating that the average oil-film thickness *h*_e_ = *O*(1 − 10^2^ μm) ≪ *R*, are consistent with the experimental observations and the assumption for the oil film to be considered as a thin surface film. In addition, the fitting value of *b* is close to −1 obtained by the scaling analysis for Eq. (9). Meanwhile, we found that using the same *a* and *b*, the Oh_e_ can also be adopted in the scaling laws proposed by other studies to fairly describe our experimental results^29,35^ (see Fig. 6e, f and ‘Methods’ for the detailed information). Overall, the proposed revised Ohnesorge number and scaling laws universally capture the jet dynamics of bubble bursting at an oil-covered aqueous surface in the current experimental range.

## Discussion

Our findings demonstrate a distinct transport mechanism related to dynamics of the bubble bursting jetting at a compound interface. Although previous studies have reported evidence of oily aerosol generation by bubble bursting at a contaminated surface^44,45^, only film droplets are considered to be responsible for the dispersion of the insoluble surface contaminants, including bacteria and aliphatic-rich hydrophobic organic species in the surface microlayer^15,19^, while the jet droplets are believed to only contribute to the transport of more soluble organic species from the underlying seawater^15,46,47^. In this study, we highlight the formation of a compound oily jet, resulting from the interplay of interfacial physicochemistry and bubble-bursting hydrodynamics. In a complete-wetting state, even surface contaminants can be dispersed by the bubble bursting jet due to the spontaneous spreading. In addition, the associated jetting dynamics will be affected by such a structurally complex interface, from the change of the effective interfacial tension as well as the viscous damping. By measuring the jet droplet compositions, our study directly evidences marine oily aerosol formation by bubble bursting jets with oil slicks, and may also serve as a valuable step to further consider the role of physicochemistry in the hydrodynamic phenomena related to bubbles. Meanwhile, although the jet radius is within 50–200 μm due to the large bubble radius in the current experiments, our scaling laws predict that the oily jet droplet size can be *O*(1–10 μm) with smaller bubble radius *R* = *O*(10^2^ − 10^3^ μm). Therefore, the compound jet droplets can potentially remain airborne for a significant amount of time, contributing to net transport of species from the bulk liquid to the atmosphere.

The results we report suggest potential environmental, biological, and public health consequences for bubble bursting in nature and industrial processes. For instance, the interface separating the ocean from Earth’s atmosphere is covered by the sea-surface microlayer, which is a physicochemically complex organic film containing significant amounts of lipids, proteins, and hydrocarbons^48,49^. The sea-surface microlayer is structurally similar to our compound interface, and our work suggests that bubble bursting can transfer the organic materials in the sea-surface microlayer by the compound jetting. With respect to the airborne disease and pollutant transmission^10,45^, our work demonstrates that the physicochemistry of the interface, such as the wettability, is required to be taken into consideration to investigate the aerosol size and droplet engulfment of the organic matter. It has been found that bacterial growth at the oil–water interface alters the wettability by secretion of biosurfactants^50^, and thus our work may shed some light on the role of the microbial-enriched, structurally complex interface on droplet erupting from bursting bubbles as well as the subsequent airborne transmission modeling. Meanwhile, bursting bubbles are considered to produce sufficient mechanical stresses to be biologically relevant to organisms living in the sea-surface microlayer, including cell damage and vegetative reproduction at the microscale^9,51,52^. Our work suggests that the presence of an insoluble viscous layer may alter the mechanical stress and energy transport during jet formation, thus facilitating smaller jet droplet production. In addition, prior work has shown that the sea-surface microlayer may even behave as a viscoelastic layer^48,53,54^, showing more complicated rheological response than the oil used in the current work. The rheological effect of the oil layer will be further considered in our future work.

In conclusion, we report that bubble bursting can disperse highly viscous surface organic matter as compound jet droplets into air, and unravel the physical mechanism related to the jetting dynamics at an oil-covered aqueous surface. We believe that our findings advance our insights into the interfacial dynamics of complex fluids, as well as the size evaluation for oily aerosol generated by bursting bubbles regarding marine environment and public health.

## Methods

### Materials

We used deionized water (resistivity = 18.2 MΩ ⋅ cm, Smart2Pure 3 UV/UF, Thermo Fisher Scientific) as the bulk liquid, and silicone oils (Sigma-Aldrich; viscosity = 5, 20, 100, 500, and 1000 cSt at 25 °C and 1 atm) as the oil layer. Their surface tensions *γ*_oa_ (or *γ*_wa_) and interfacial tension *γ*_ow_ were measured using the pendant-drop method, and their density *ρ* and viscosity *μ* are listed in Table 1.

### High-speed imaging

A square transparent acrylic container with a cross-section area of 120 × 120 mm^2^ and a height of 40 mm was designed to hold the liquids (Fig. 1a). A stainless-steel needle (18 Gauge) was used to generate the gas bubble by a syringe pump (11 Pico Plus Elite, Harvard Apparatus). The distance between the water surface and the needle was set as 5 mm, and an oil layer with a thickness of *h* was deposited onto the water surface using a pipette. The cavity collapse and jet formation were recorded synchronously from the side view by two high-speed cameras (FASTCAM Mini AX200, Photron) mounted with focus lenses using a magnification of 1.8–2.3, illuminated by two LED panels, respectively. We used a frame rate of 10,000 frames per second, an exposure time of 20–50 μs, and an image resolution of 1024 × 762 pixels. The obtained images were further processed using Fiji (ImageJ)^55^.

### Viscous damping of the capillary waves at an oil-covered aqueous surface

We consider the damping rate of the surface capillary waves in a system that consists of a thin layer of oil with a finite thickness, *h*_e_, floating on water with an infinite depth, while the bubble cavity curvature is neglected (Fig. 6d). For a capillary wave with a wave number *k*, the boundary layer thickness in the oil layer is^38^

14 $${h}_{{{{{{\mathrm{bo}}}}}}}=\sqrt{\frac{2{\mu }_{{{{{{\mathrm{o}}}}}}}}{\omega {\rho }_{{{{{{\mathrm{o}}}}}}}}},$$

where the capillary frequency

15 $$\omega =\sqrt{\frac{{\gamma }_{{{{{{\mathrm{e}}}}}}}{k}^{3}}{{\rho }_{{{{{{\mathrm{w}}}}}}}}}.$$

Regarding the dominant capillary wave with *k* ~ *R*^−1^ in our bubble bursting experiments, *h*_bo_ is calculated to *O*(1 mm), while the observed thickness of the oil layer on the bubble cavity surface is much smaller than the bubble radius. Given that *h*_bo_ ≫ *h*_e_, the oil layer can be considered as a viscous surface film, with two-dimensional shear viscosity *η*_1_ = *μ*_o_*h*_e_ and two-dimensional dilatational viscosity *η*_2_ = 3*μ*_o_*h*_e_^38^. Additionally, we estimate boundary layer thickness in water as^56^

16 $${h}_{{{{{{\mathrm{bw}}}}}}}=\sqrt{\frac{2{\mu }_{{{{{{\mathrm{w}}}}}}}}{\omega {\rho }_{{{{{{\mathrm{w}}}}}}}}},$$

and we obtain *kh*_bw_ ≪ 1 and *kh*_w_ ≫ 1, where *h*_w_ is the water depth in the experiments. Therefore, we can adopt a deep water assumption. From the previous studies of a thin floating fluid layer^38,56^, the total dissipation rate of the capillary waves is written as

17 $$\langle D\rangle =\frac{4{\mu }_{{{{{{\mathrm{w}}}}}}}{k}^{2}}{{\rho }_{{{{{{\mathrm{w}}}}}}}}\frac{2+\zeta }{2+2\zeta +{\zeta }^{2}}{E}_{0}+{\left(\frac{1}{2}\frac{{\mu }_{{{{{{\mathrm{w}}}}}}}\omega {k}^{2}}{{\rho }_{{{{{{\mathrm{w}}}}}}}}\right)}^{\frac{1}{2}}\frac{2\zeta +{\zeta }^{2}}{2+2\zeta +{\zeta }^{2}}{E}_{0}.$$

Here 〈〉 implies an average over one cycle of the oscillation, *E*_0_ is the total energy of wave oscillation, and *ζ* is a dimensionless number comparing the effect of surface viscosity and the bulk water viscosity as

18 $$\zeta ={\left(\frac{1}{2}{\mu }_{{{{{{\mathrm{w}}}}}}}{\rho }_{{{{{{\mathrm{w}}}}}}}\omega \right)}^{-\frac{1}{2}}{k}^{2}({\eta }_{1}+{\eta }_{2})=4{\left(\frac{1}{2}{\mu }_{{{{{{\mathrm{w}}}}}}}{\rho }_{{{{{{\mathrm{w}}}}}}}\omega \right)}^{-\frac{1}{2}}{k}^{2}{\mu }_{{{{{{\mathrm{o}}}}}}}{h}_{{{{{{\mathrm{e}}}}}}}.$$

The first term on the right-hand side of Eq. (17) is the dissipation rate from the irrotational motion and the interaction between the irrotational motion and the water boundary layer motion, while the second term is the dissipation rate from the rotational motion and shear flow in the surface layer. In our experiments, *ζ* ≪ 1. Thus, we obtain a leading order approximation for the total dissipation rate as

19 $$\langle D\rangle =\frac{4({\mu }_{{{{{{\mathrm{w}}}}}}}+{\mu }_{o}k{h}_{{{{{{\mathrm{e}}}}}}}){k}^{2}}{{\rho }_{{{{{{\mathrm{w}}}}}}}}{E}_{0}.$$

### Alternative scaling laws for the oily jetting

Considering the energy balance during cavity collapse, the surface energy of the bubble collapse, minus the viscous dissipation associated to the motion induced by the capillary wave, provides the surplus of the mechanical energy forming the jet^27,35^. Using a balance of the inertia, capillarity and viscosity effects at the onset of the jet ejection, the following scaling laws using Oh have been obtained as^35,40^*r*_j_/*R* = *k*_*d*_(0.043−Oh)^5/4^Oh^−1/2^ and *v*_j_/*v*_c_ = *k*_*v*_(1+2.2Bo)^−3/4^(0.043−Oh)^−3/4^Oh^1/2^, where *k*_*d*_ and *k*_*v*_ are prefactors depending on the measurement position with Bo < 1 and Oh < Oh_c_^27,29^. Therefore, with the revised Ohnesorge number Oh_e_ < Oh_c_, we can obtain

20 $$\frac{{r}_{j}}{R}={k}_{d}{(0.043-{{{{{\mathrm{O}}}}}}{{{{{{\mathrm{h}}}}}}}_{{{{{{\mathrm{e}}}}}}})}^{5/4}{{{{{\mathrm{O}}}}}}{{{{{{\mathrm{h}}}}}}}_{{{{{{\mathrm{e}}}}}}}^{-1/2},$$

21 $$\frac{{v}_{{{{{{\mathrm{j}}}}}}}}{{v}_{{{{{{\mathrm{c}}}}}}}}={k}_{v}{(1+2.2{{{{{\mathrm{Bo}}}}}})}^{-3/4}{(0.043-{{{{{\mathrm{O}}}}}}{{{{{{\mathrm{h}}}}}}}_{{{{{{\mathrm{e}}}}}}})}^{-3/4}{{{{{\mathrm{O}}}}}}{{{{{{\mathrm{h}}}}}}}_{{{{{{\mathrm{e}}}}}}}^{1/2}.$$

Figure 6e, f shows that Eqs. (20) and (21) can also capture our experiment results well, with *k*_*d*_ = 0.6 and *k*_*v*_ = 12.6, which are similar with the literature values for the moment where the jet tip reaches the undisturbed water level^35,40^. This verifies that the revised Ohnesorge number Oh_e_ we proposed can be adopted as the dominant dimensionless number characterizing the bursting dynamics of gas bubbles at an oil-covered aqueous surface.

## Supplementary information

Supplementary information.

Description of Additional Supplementary Files.

Supplementary Movie 1.

Supplementary Movie 2.

Supplementary Movie 3.

Supplementary Movie 4.

Supplementary Movie 5.

## Data Availability

The data that support the findings of this study are available from the corresponding author upon reasonable request.
