# Supplementary material for: Compound jetting from bubble bursting at an air-oil-water interface
Source: Nat Commun. 2021 Nov 2;12:6305. doi: 10.1038/s41467-021-26382-w (PMC8563946; doi:10.1038/s41467-021-26382-w)
Supplement: Supplementary file 1 — Supplementary information. [file 41467_2021_26382_MOESM1_ESM.pdf]

Supplementary Information: Compound jetting from bubble bursting at an air-oil-water interface

Bingqiang Ji[1*], Zhengyu Yang[1*], and Jie Feng[1,2†]

[1]*Department of Mechanical Science and Engineering, University of Illinois at Urbana–Champaign, Urbana, IL, 61801, USA.*

[2]*Materials Research Laboratory, University of Illinois at Urbana–Champaign, Urbana, IL, 61801, USA.*

[*]*These authors contributed equally to this work.*

[†]*Email: jiefeng@illinois.edu.*

Supplementary Discussion

Regime of no jet drop. A regime of no jet drop from the bursting bubble occurs when the oil layer thickness h is higher than a critical value $h_c \approx 1.2R$, where R is the bubble radius. For the bubble with a radius $R = 1.67$ mm initially resting at the water surface covered by an oil layer with a thickness of $h \gtrsim 1.2R$, the water film ruptures before the oil layer, showing a film retraction pattern similar to bursting of a fluid film in a viscous environment [1] (Supplementary Figure 1 and Supplementary Movies 1 and 2). The bubble is then trapped in the oil layer, and no jet droplets are observed even when the trapped bubble bursts (Supplementary Movie 3).

Dumbbell-shaped droplets. We observe that dumbbell-shaped droplet forms when two successively ejected oily jet drops collide and partially merge, as shown in Supplementary Figure 2 and Supplementary Movie 4. After the bubble bursts at the water surface covered with 100 cSt silicone oil of a layer thickness $h = 0.8R$, the third and fourth jet drops collide, resulting in a partially merged droplet that remains a dumbbell-like shape as it falls, rebounds, and rotates for more than dozens of milliseconds until it enters the oil-covered surface. Previous experiments show that the clean water drops typically coalesce or rebound off each other in milliseconds [2, 3], so our observation indicate that a thin oil film may exist on the jet droplets surface that prevent the droplet coalescence.

Jet droplet compositions. In order to estimate the compositions of the ejected jet droplets, we added 6 μm poly(methyl methacrylate) (PMMA) particles with a concentration of 0.1 wt% into the oil layer, and measured the particle concentration in the top jet droplet for oil composition quantification. The top jet droplets ejected by bubble bursting were collected using glass slides, and the jet droplet ejection process was captured by the high-speed camera. We confirmed that the particles in the oil layer cannot enter the water in the bubble bursting experiments. The top jet droplet radius was measured to obtain its volume, which is consistent with the value without particles. The collected oily jet droplets were dried in the oven at 85 °C on the glass slide. Then the deposited particles were imaged by a microscope (Nikon ECLIPSE Ti2, Nikon) with a 20× objective lens, as shown in Supplementary Figure 3(a). The particle number N was counted by image analysis, as shown in Supplementary Figure 3(b). Then, the oil volume ratio in the top jet droplet ϕ_o is estimated as $\phi_o = C/(eC_0)$, where C and C_0 are the particle number concentrations in the collected top jet droplet and the oil layer, respectively, and e is the enrichment factor of particles in the top jet droplet. In prior work [4–7], the dispersed particles will be enriched by jet droplets from the cavity collapse, so we consider this enrichment factor in our estimation. Here, we choose $e = 10$ according to the literature value for 50-200 μm jet droplets [4, 5]. Supplementary Figure 3(c) shows the dependence of ϕ_o on the dimensionless oil layer thickness h/R. For 5 cSt silicone oil, ϕ_o is estimated to be near 1 which means that the top jet droplet consists of mainly oil. In addition, ϕ_o further decreases with oil viscosity, which is consistent with our argument that highly viscous oil spreads slower, and therefore less oil would reach the jet. For 500 cSt and 1000 cSt silicone oils, the particles were not observed in the top jet droplet, indicating that the oil fraction was very small and beyond the detection limit of the current method. Therefore, they are not shown in Supplementary Figure 3(c).

Top jet droplet radius and velocity. The radius r_d and velocity v_d of the top jet droplet at the moment of detachment were measured, as shown in Supplementary Figure 4. The jet droplet radius and velocity show similar trends with the thickness and viscosity of the oil layer as the jet tip radius and velocity. We find that r_d is approximately once or twice as large as r_j, consistent with previous numerical study [8] which reported that r_d can be far larger than r_j due to the spatio-temporal growth of the upward ballistic jet before break-up.

Jet droplet number and jetting height. We find that the number of jet droplets and the jetting height increase with the oil viscosity and oil layer thickness as shown in Supplementary Movie 5. The jetting height is defined as the maximum height of the jet before the retraction occurs, relative to the undisturbed air-oil interface.

Spreading of macroscopic oil edges tracked by particles. To clearly visualize the oil spreading along the cavity surface, experiments were conducted by dispersing 6 μm PMMA particles into the oils with a concentration of 0.05-0.5 w/v% to track the position of the macroscopic oil edges, as shown in Supplementary Figure 5. The jet radius and velocity were verified to be unaffected by the tracer particles. In addition, the position of the oil edges tracked by particles at the cavity surface in Supplementary Figures 5(c-e) is similar to that observed in Figures 3(b-d) without particle in the main text. It shows that the propagation of the macroscopic oil edge is slower with increasing oil viscosity (Supplementary Figures 5b-d) and basically unaffected by the oil layer thickness (Supplementary Figures 5d-e).

Effect of the substrate liquid viscosity. To further demonstrate the role of oil spreading dynamics, we preformed additional experiments using a viscous liquid of 40 w/w% glycerin-water solution (Supplementary Table I) with a 20 cSt silicone oil layer. The glycerin solution has a viscosity of 4.1 times as the water viscosity, corresponding to a larger t_s (time scale for oil spreading on the bubble cavity surface) and a similar t_c (time scale for cavity collapse). The produced jet tip radius r_j and velocity v_j are shown in Supplementary Figure 6. In this case, since we increase t_s, the oil will spread slower and may not reach the cavity nadir. Therefore, the existence of oil layer is not expected to significantly change r_j and v_j. As shown in Supplementary Figure 6, with a water substrate, the oil layer largely decreases r_j and increases v_j; while with an aqueous glycerin solution substrate, r_j and v_j are barely changed. The results are consistent with our argument that a more viscous substrate liquid might weaken the effect of the oil layer on the jet dynamics, presumably due to the slower spreading of the oil film (larger t_s) on the cavity surface.

Supplementary Table I: Physical properties of the liquids used in the experiments (o: oil; w: aqueous solution; a: air).

Liquids	ρ (kg/m^3)	μ (mPa·s)	γ_{wa} (mN/m)	γ_{oa} (mN/m)	γ_{ow} (mN/m)
40 w/w% glycerin-water solution	1099	3.72	70.1 ± 1.9	N/A	N/A
20 cSt silicone oil	950	19	N/A	19.4 ± 0.7	30.3 ± 1.0

Supplementary Figures

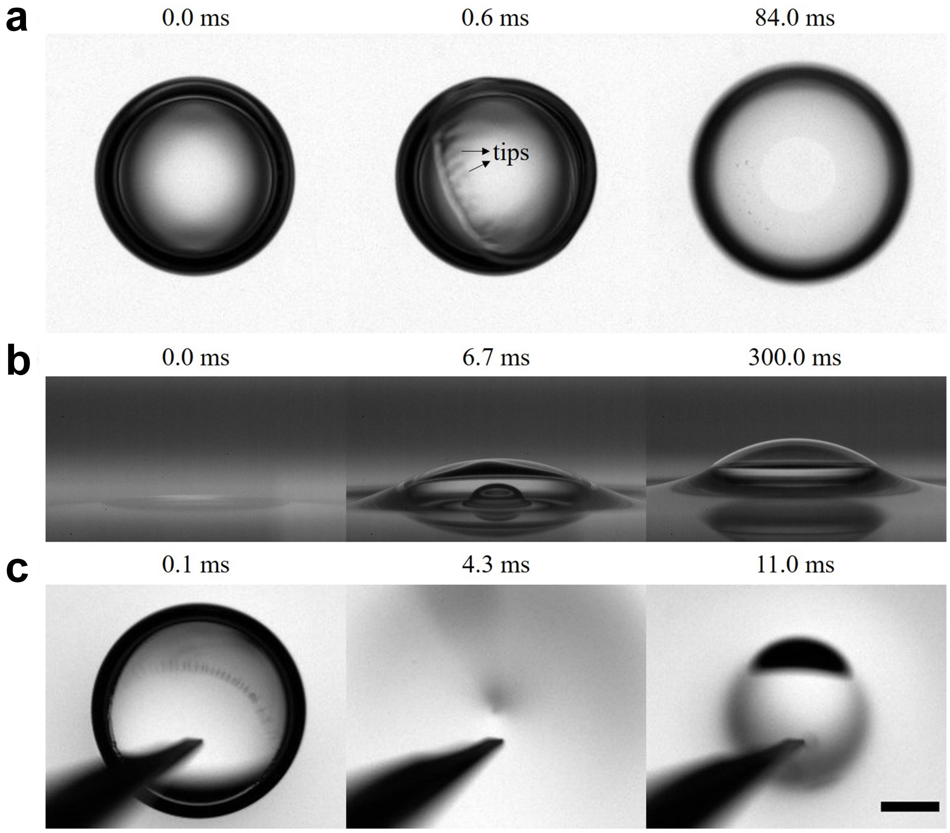

Supplementary Figure 1: Experimental images of no jet drop regime. **(a)** Top view of a bubble entering the layer of 5 cSt silicone oil with a dimensionless layer thickness $h/R = 1.2$ ($R = 1.67$ mm). The rupturing water film in the bubble cap shows a fingering pattern. See also Supplementary Movie 1. **(b)** Side view of the bubble entering the oil layer. After the water film ruptures, a water jet producing no droplets is formed inside the bubble, and the bubble enters the oil layer with its position elevated. See also Supplementary Movie 2. **(c)** Top view of the bubble bursting in the oil layer. No jet drops are produced when it is punctured. See also Supplementary Movie 3. $t = 0$ is defined as **(a)(b)** the start of the water film rupture and **(c)** the start of the oil film rupture. The scale bar represents 1 mm.

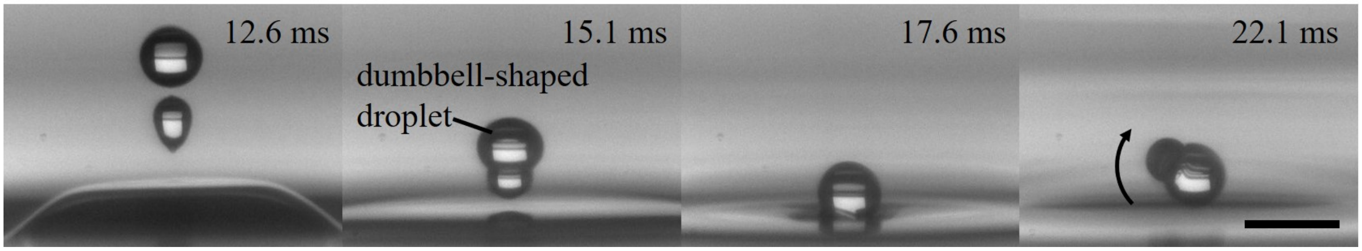

Supplementary Figure 2: Side view of the formation of a dumbbell-shaped jet droplet. Two successively ejected jet droplets partially merge and form a dumbbell-shaped droplet. The dumbbell-shaped droplet keeps its shape while it rebounds off from the liquid surface and rotates. The scale bar represents 1 mm. The oil viscosity is 100 cSt and the dimensionless layer thickness is $h/R = 1.2$ ($R = 1.67$ mm). See also Supplementary Movie 4. $t = 0$ is defined as the start of the bubble cap rupture.

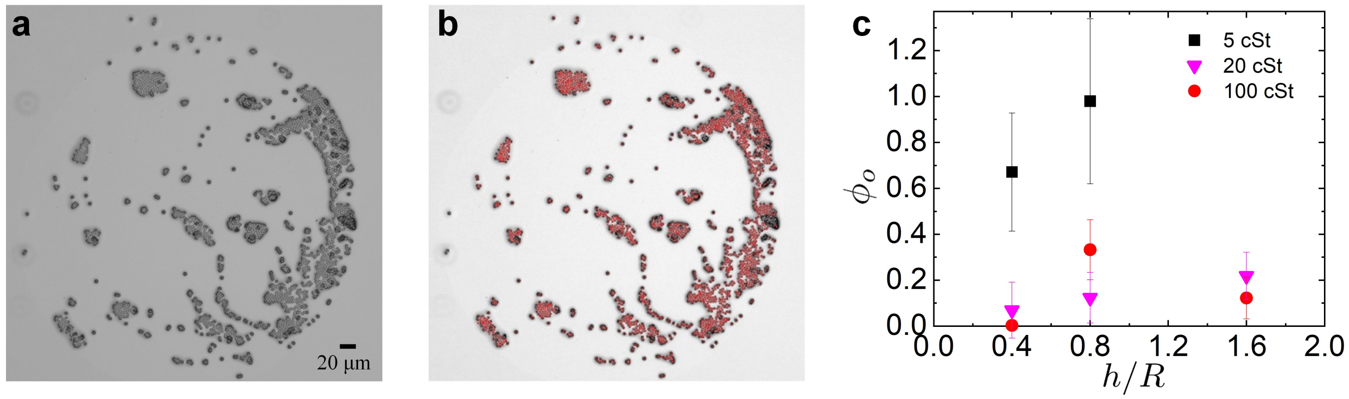

Supplementary Figure 3: Measurement of jet droplet compositions. Original image (**a**) and processed image with the particles identified by red dots (**b**) of the dried top jet droplet from bubble bursting at the aqueous surface covered by 5 cSt silicone oil layer with 6 μm PMMA particles, with $h/R = 0.4$ and particle concentration of 0.1 wt%. (**c**) Variation of the oil volume ratio ϕ_o in the top jet droplet by bubble bursting at an oil-covered aqueous surface with dimensionless oil layer thickness h/R. Error bars are calculated as the standard deviations of data of at least 5 runs.

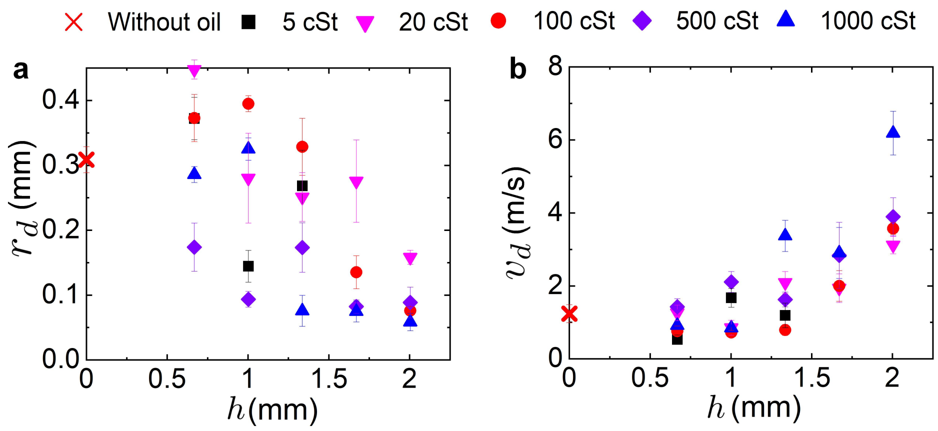

Supplementary Figure 4: Top jet droplet radius and velocity. Dependencies of the (**a**) radius r_d and (**b**) velocity v_d of the top jet droplet with the oil layer thickness h for different oil viscosities. r_d and v_d are measured when the top jet droplet detaches. Error bars are calculated as the standard deviations of data of at least 10 runs.

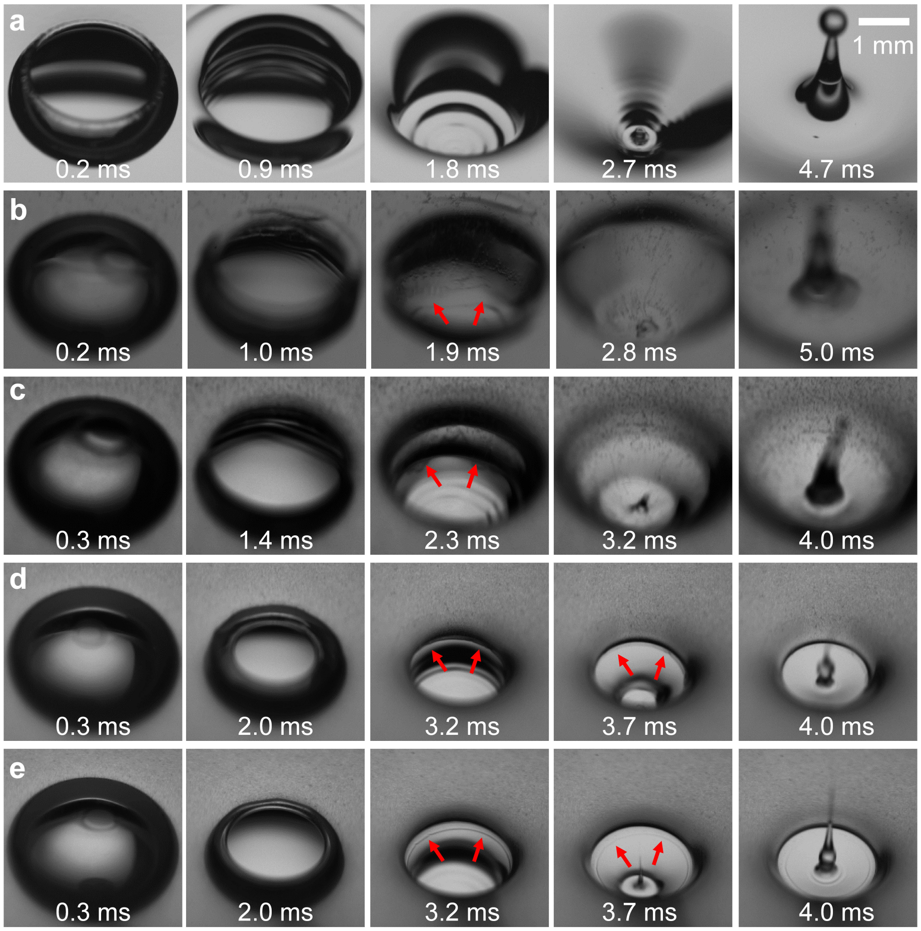

Supplementary Figure 5: Top-view snapshots of oil spreading on the cavity surface tracked by particles during bubble bursting at the aqueous surface. The water surface shown here are (**a**) clean and covered by a layer of (**b**) 5 cSt silicone oil, $h/R = 0.8$, particle concentration in oil $\phi_p = 0.05$ wt%; (**c**) 100 cSt silicone oil, $h/R = 0.8$, $\phi_p = 0.5$ wt%; (**d**) 1000 cSt silicone oil, $h/R = 0.8$, $\phi_p = 0.5$ wt%; and (**e**) 1000 cSt silicone oil, $h/R = 1.2$, $\phi_p = 0.5$ wt%. The scale bar represents 1 mm. In order to clearly visualize the oil spreading along the cavity surface, 6 μm PMMA particles were added into the oils to track the macroscopic oil edges. Compared with the pure water case (**a**), a macroscopic oil edge (marked by red arrows) along the cavity after the cap rupture at an oil-covered surface is clearly observed, which is generated by oil spreading in the complete wetting state (**b-e**).

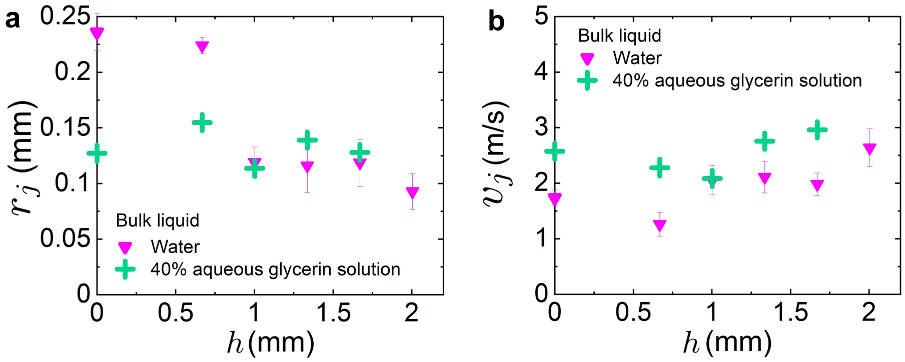

Supplementary Figure 6: Effect of the substrate liquid viscosity on jet dynamics. Dependencies of (**a**) the jet tip radius r_j and (**b**) the jet tip velocity v_j on the oil layer thickness h for bubble bursting at a water (triangles) or aqueous glycerin solution (40 w/w%, crosses) surfaces covered by a 20 cSt silicone oil layer. Bubble radius $R = 1.6$ mm. r_j and v_j are measured when the jet tip crosses the undisturbed air-oil interface. Error bars are calculated as the standard deviations of data of at least 10 runs.

Supplementary References

[1] Reyssat, É. & Quéré, D. Bursting of a fluid film in a viscous environment. *Europhys. Lett.* **76**, 236 (2006).

[2] Jiang, Y., Umemura, A. & Law, C. An experimental investigation on the collision behaviour of hydrocarbon droplets. *J. Fluid Mech.* **234**, 171–190 (1992).

[3] Qian, J. & Law, C. K. Regimes of coalescence and separation in droplet collision. *J. Fluid Mech.* **331**, 59–80 (1997).

[4] Blanchard, D. C. The ejection of drops from the sea and their enrichment with bacteria and other materials: a review. *Estuaries* **12**, 127–137 (1989).

[5] Sakai, M., Tanaka, A., Egawa, H. & Sugihara, G. Enrichment of suspended particles in top jet drops from bursting bubbles. *J Colloid Interf. Sci.* **125**, 428–436 (1988).

[6] Chingin, K., Yan, R., Zhong, D. & Chen, H. Enrichment of surface-active compounds in bursting bubble aerosols. *Acs Omega* **3**, 8709–8717 (2018).

[7] Burger, S. R. & Bennett, J. Droplet enrichment factors of pigmented and nonpigmented serratia marcescens: possible selective function for prodigiosin. *Applied and environmental microbiology* **50**, 487–490 (1985).

[8] Blanco-Rodríguez, F. J. & Gordillo, J. On the sea spray aerosol originated from bubble bursting jets. *J. Fluid Mech.* **886** (2020).
