# Supplementary material for: Compound jetting from bubble bursting at an air-oil-water interface
Source: Nat Commun. 2021 Nov 2;12:6305. doi: 10.1038/s41467-021-26382-w (PMC8563946; doi:10.1038/s41467-021-26382-w)
Supplement: Supplementary file 2 — Description of Additional Supplementary Files. [file 41467_2021_26382_MOESM2_ESM.pdf]

Description of Additional Supplementary Files

File Name: Supplementary Movie 1
Description: Top view of a bubble with R = 1.67 mm entering the 5 cSt silicone oil layer with h/R = 1.2 above the water surface.

File Name: Supplementary Movie 2
Description: Side view of a bubble with R = 1.67 mm entering the 5 cSt silicone oil layer with h/R = 1.2 above the water surface.

File Name: Supplementary Movie 3
Description: Top view of a bubble with R = 1.67 mm bursting in a 5 cSt silicone oil layer with h/R = 1.2 above the water surface by puncturing with a sharp needle.

File Name: Supplementary Movie 4
Description: Side view of the formation and motion of the dumbbell-shaped droplet after bubble bursting at the 100 cSt silicone oil-covered water surface with h/R = 0.8.

File Name: Supplementary Movie 5
Description: Side view of jet droplets formation by bubble bursting at an oil-covered water surface. From left to right, the water surface is covered by 5 cSt silicone oil with h/R = 0.4, 1000 cSt silicone oil with h/R = 0.4, and 1000 cSt silicone oil with h/R = 1.2.
